# Prevalence and Clinical Characteristics of Dizziness, Imbalance, and Associated Factors Following Bariatric Surgery

**DOI:** 10.3390/jcm15062265

**Published:** 2026-03-17

**Authors:** Sumaia Alanazi, Murad Almomani, Abdullah S. Alanazi, Abdullah A. Albarrak, Danah Alyahya, Salam M. Almomani, Esraa M. Almomani, Yassin Abdelsamad, Shagun Agarwal, Faizan Kashoo

**Affiliations:** 1 ENT Department, King Abdulaziz University Hospital, King Saud University, Riyadh 12629, Saudi Arabia; susalanazi@ksu.edu.sa (S.A.); mualmomani@ksu.edu.sa (M.A.); 2 Public Health Intelligence, Saudi Public Health Authority, Riyadh 13351, Saudi Arabia; asanazi@pha.gov.sa; 3 Department of Surgery, College of medicine, Majmaah University, Al-Majmaah 11952, Saudi Arabia; aa.barrak@mu.edu.sa; 4 Department of Physical Therapy and Health Rehabilitation, College of Applied Medical Sciences, Majmaah University, Al-Majmaah 11952, Saudi Arabia; f.kashoo@mu.edu.sa; 5 Faculty of Medicine, Jordan University of Science and Technology, Irbid 22110, Jordan; salamalmomani2002@gmail.com (S.M.A.); almomaniesraa03@gmail.com (E.M.A.); 6 MEDEL Research Department, Riyadh 11563, Saudi Arabia; yassin.samad@medel.com; 7 School of Allied Health Sciences, Galgotias University, Greater Noida 203201, India; shagunmpt@gmail.com

**Keywords:** bariatric surgery, dizziness, vertigo, balance disorders, weight loss, post-operative complications

## Abstract

**Background/Objectives**: Bariatric surgery has emerged as an effective intervention for severe obesity; however, post-operative dizziness remains poorly characterized in the literature. This study aimed to determine the prevalence of dizziness, imbalance, and hearing problems following bariatric surgery and to identify associated risk factors. **Methods**: A cross-sectional study was conducted among 156 patients who underwent bariatric surgery at multiple centers in Saudi Arabia. Data were collected through structured questionnaires assessing demographic characteristics, surgical details, and post-operative vestibular symptoms. Bivariate and multivariate logistic regression analyses were performed to identify predictors of dizziness. **Results**: The prevalence of post-operative dizziness was 77.3% (95% CI: 70.0–83.3%), imbalance was 38.0% (95% CI: 30.6–46.0%), and hearing problems were 10.7% (95% CI: 6.7–16.6%). Bivariate logistic regression identified weight loss was significantly associated with dizziness (OR = 1.063, 95% CI: 1.024–1.103, *p* = 0.001). In the multivariate model, each percentage point increase in weight loss was associated with a 6.1% increased dizziness (adjusted OR = 1.061, 95% CI: 1.017–1.107, *p* = 0.006). Dizziness was strongly associated with imbalance (chi-square = 14.325, *p* < 0.001) and falls (chi-square = 7.085, *p* = 0.008). **Conclusions**: Vestibular complications, particularly dizziness, are highly prevalent following bariatric surgery and demonstrate a significant dose–response relationship with the magnitude of weight loss. Enhanced awareness and systematic screening for dizziness in post-bariatric patients are warranted.

## 1. Introduction

The global epidemic of obesity represents one of the most significant public health challenges of the twenty-first century, with prevalence rates exceeding 39.7% in many developed nations [1] and rising rapidly to 36.5% in developing economies like Saudi Arabia [2]. Bariatric surgery has emerged as the most effective intervention for severe obesity (BMI ≥ 40 kg/m^2^ or ≥35 kg/m^2^ with comorbidities), achieving sustained weight loss of 25–35% and substantial improvements in obesity-related comorbidities including type 2 diabetes, hypertension, and obstructive sleep apnea [3,4]. The number of bariatric procedures performed annually has increased dramatically, with an estimated 900,000 operations conducted in the United States from 2016–2020 [5] and 15,000 surgeries annually in Saudi Arabia [6]. Emerging minimally invasive metabolic interventions, including endovascular bariatric procedures, further underscore that bariatric strategies extend beyond weight reduction alone and may induce systemic physiological effects with implications beyond metabolic outcomes [7].

Despite the metabolic benefits, bariatric surgery induces profound physiological changes that may affect multiple organ systems. The gastrointestinal tract undergoes structural reconfiguration, nutrient absorption is altered, and patients experience rapid changes in body composition, hormonal profiles, and hemodynamic parameters [8,9,10]. These alterations, while contributing to weight loss and metabolic improvement, may also precipitate unintended complications that impact quality of life and functional capacity [11]. Post-operative dizziness following bariatric surgery refers to a subjective disturbance of spatial orientation reported during the post-operative period and may include sensations of lightheadedness, pre-syncope, disequilibrium, or vertigo [12]. In this population, post-operative dizziness is typically multifactorial rather than attributable to a single vestibular disorder. Proposed contributors include dehydration and orthostatic hypotension [13] related to rapid weight loss and autonomic changes, hypoglycemia during early dietary adaptation, dumping syndrome, micronutrient deficiencies and anemia due to altered absorption, as well as transient effects of anesthesia in the immediate post-operative phase [14].

The pathophysiological mechanisms linking bariatric surgery to post-operative dizziness are multifactorial and incompletely understood [15]. Rapid weight loss may alter the mechanical properties of the vestibular apparatus, including changes in endolymphatic fluid dynamics and otolithic membrane function [16]. Nutritional deficiencies, particularly of vitamin B12, vitamin D, and iron, are common following bariatric surgery and may affect neural conduction [17]. Additionally, postural hypotension resulting from altered baroreceptor sensitivity and reduced cardiac preload may contribute to orthostatic dizziness [18].

Despite these plausible mechanisms, the prevalence and clinical characteristics of post-operative dizziness following bariatric surgery remain poorly characterized. Existing literature consists primarily of case reports and small case series, with limited systematic evaluation of symptom prevalence, temporal patterns, and risk factors. This knowledge gap has important clinical implications, as post-operative dizziness may lead to falls, injuries, reduced mobility, and impaired quality of life in a population already at increased risk for musculoskeletal complications [19]. The primary objective of this study was to determine the prevalence of post-operative dizziness, imbalance, and hearing problems in a cohort of patients who had undergone bariatric surgery. Secondary objectives included characterizing the temporal onset and clinical features of these symptoms, identifying demographic and clinical risk factors, and evaluating associations between post-operative dizziness and functional outcomes including falls. We hypothesized that post-operative dizziness would be common following bariatric surgery and would demonstrate associations with the magnitude of weight loss and nutritional status.

## 2. Materials and Methods

### 2.1. Subjects

This cross-sectional study was conducted among patients who had undergone bariatric surgery at multiple centers across Saudi Arabia between 2018 and 2024. Participants were recruited through bariatric surgery clinics, patient support groups, and social media platforms dedicated to post-bariatric patients. This recruitment strategy, while facilitating access to a diverse sample, introduces important methodological considerations. Recruitment through specialized clinics and support groups may have preferentially captured patients experiencing ongoing post-operative concerns. Similarly, social media recruitment may have attracted symptomatic individuals motivated to participate in research addressing their health issues. These factors may have contributed to an overrepresentation of symptomatic individuals, potentially inflating the observed prevalence beyond what would be expected in an unselected population. Inclusion criteria required participants to be at least 18 years of age and to have undergone either gastric sleeve or gastric bypass surgery at least one month prior to enrollment. No exclusion criteria were applied based on pre-existing medical conditions or concurrent medications. The self-reported nature of symptom assessment introduces response bias, whereby symptomatic patients may be more likely to recall and report symptoms. Additionally, the broad definition of dizziness (self-reported without clinical differentiation) may capture heterogeneous symptoms including true vestibular dizziness, orthostatic lightheadedness, medication-related symptoms, and other non-vestibular causes, limiting our ability to distinguish vestibular pathology from other postural symptoms.

### 2.2. Survey Tool

Data were collected through a structured, self-administered questionnaire developed specifically for this study. The questionnaire captured demographic information (age, gender, geographic location), surgical details (procedure type, date of surgery, pre-operative weight, lowest post-operative weight), and post-operative experiences (supplement use, side effects, complications). The primary outcomes of interest were self-reported dizziness, imbalance/instability, and hearing problems occurring after surgery. For participants reporting dizziness, additional details were collected regarding timing of onset, episode duration, frequency, associated symptoms, and functional impact.

The questionnaire was pilot-tested to evaluate its reliability and accuracy among 10 participants. This study relied exclusively on self-reported symptom assessment without objective vestibular testing (videonystagmography, caloric testing, or posturography). The absence of objective measures precludes definitive characterization of vestibular function and limits our ability to distinguish peripheral vestibular dysfunction from central causes, orthostatic intolerance, or other non-vestibular etiologies. The reliability was assessed using the Cronbach’s alpha coefficient yielding a score of 0.91, while content validity was confirmed by mutual agreement among the panel of expert surgeons, audiologists, and researchers specializing in vestibular disorders.

### 2.3. Statistical Analysis

Descriptive statistics were calculated for all variables, with continuous variables presented as mean ± standard deviation and categorical variables as frequencies and percentages. The prevalence of vestibular symptoms was calculated with 95% confidence intervals using the Wilson score method. Bivariate associations between categorical variables were assessed using chi-square tests or Fisher’s exact test as appropriate. For chi-square analyses, effect sizes (Cramér’s V) were calculated, and 95% confidence intervals were derived to provide a measure of precision and strength of association. Continuous variables were compared between groups using independent t-tests or Mann–Whitney U tests based on distributional assumptions. Of the 156 participants enrolled in this study, six (3.8%) had incomplete responses for all vestibular symptom questions and were excluded from prevalence calculations, resulting in *N* = 150 for symptom prevalence analyses. For logistic regression analysis, 20 participants (12.8%) had missing pre-operative or post-operative weight data, precluding calculation of weight loss percentage. An additional six participants had missing time-since-surgery data. Complete case analysis for all regression variables (dizziness outcome, age, gender, weight loss percentage, supplement use, and time since surgery) resulted in *N* = 136 participants. Vitamin D deficiency was not included in the regression analysis due to missing laboratory data for 68 participants.

Bivariate logistic regression analyses were conducted to identify unadjusted predictors of dizziness, with results expressed as odds ratios (OR) and 95% confidence intervals (CI). Multivariate logistic regression was performed to assess independent associations while adjusting for potential confounders including age, gender, supplement use, and time since surgery. Spearman rank correlation coefficients were calculated to examine relationships between continuous variables. Statistical significance was set at *p* < 0.05 for all analyses. Data were analyzed using Python (version 3.12) with the SciPy and Statsmodels packages.

## 3. Results

### 3.1. Subject Characteristics:

A total of 156 patients who underwent bariatric surgery were enrolled in this cross-sectional study. The demographic and clinical characteristics of the study population are presented in Table 1. The mean age of participants was 36.9 ± 9.9 years (range: 18–65 years), with the majority being female (*n* = 101, 64.7%). The cohort was predominantly from Riyadh (*n* = 97, 62.2%), with representation from multiple regions across Saudi Arabia.

The vast majority of participants underwent gastric sleeve surgery (*n* = 144, 92.3%), while a smaller proportion received gastric bypass procedures (*n* = 7, 4.5%). The mean pre-operative weight was 119.5 ± 24.6 kg, with participants achieving a mean lowest post-operative weight of 74.9 ± 17.4 kg, corresponding to an average weight loss of 36.2 ± 12.5%. Regarding the temporal distribution, 42.9% of participants (*n* = 67) were within the first year post-surgery, 30.1% (*n* = 47) were 1–3 years post-surgery, and 23.1% (*n* = 36) were beyond 3 years from their surgical procedure (Table 1).

### 3.2. Prevalence of Post-Operative Vestibular Symptoms

Dizziness was the most commonly reported symptom, affecting 116 of 150 respondents. This prevalence should be interpreted cautiously, as recruitment strategy and self-reported methodology may have contributed to overrepresentation of symptomatic individuals and inclusion of non-vestibular causes (77.3%; 95% CI: 70.0–83.3%). Imbalance and instability were reported by 57 of 150 participants (38.0%; 95% CI: 30.6–46.0%), while hearing problems were less prevalent, occurring in 16 of 150 respondents (10.7%; 95% CI: 6.7–16.6%). Notably, 31 participants (20.7%) reported experiencing falls directly attributable to post-operative imbalance. Among those reporting dizziness, 54 of 116 (46.6%) also experienced concurrent imbalance, indicating substantial symptom overlap (Table 2).

### 3.3. Characteristics of Dizziness Episodes

Among the 116 participants who reported post-operative dizziness, detailed characteristics of their symptoms were analyzed (Table 3). The onset of dizziness was predominantly early in the post-operative course: 35.3% (*n* = 41) experienced their first episode within 1–3 weeks following surgery, while an additional 19.0% (*n* = 22) reported onset between 2–6 months post-operatively. The duration of dizziness episodes was typically brief, with 81.0% (*n* = 94) reporting episodes lasting only seconds, and 15.5% (*n* = 18) experiencing episodes of 2–5 min.

**Figure 1:**
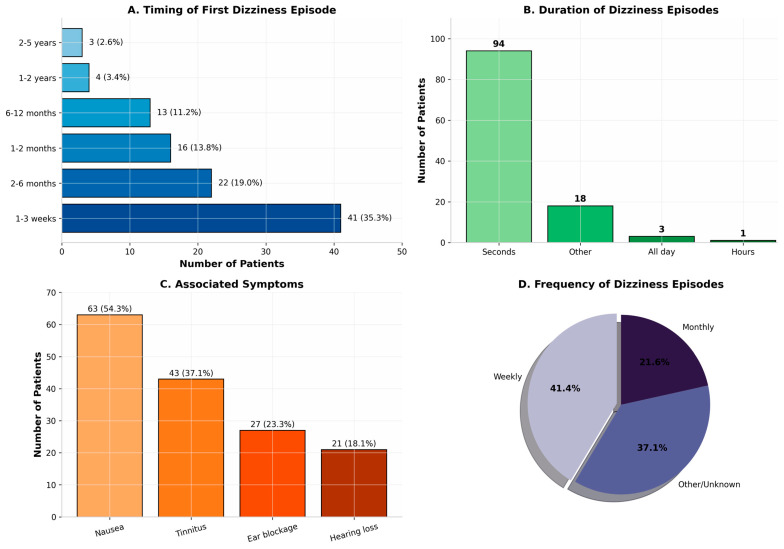
Characteristics of Dizziness Episodes in Affected Participants (*n* = 116).

The frequency of episodes was considerable: 41.4% experienced weekly episodes, 37.1% reported daily occurrences, and 21.6% experienced monthly episodes. Associated symptoms were common among dizzy patients. Nausea accompanied dizziness in 54.3% of cases (*n* = 63), tinnitus in 37.1% (*n* = 43), ear blockage in 23.3% (*n* = 27), and hearing loss in 18.1% (*n* = 21) (Figure 1).

### 3.4. Bivariate and Multivariate Regression Analyses

To identify predictors of post-operative dizziness, we conducted bivariate logistic regression analyses for each potential risk factor (Table 4). Among the variables examined, only weight loss percentage demonstrated a statistically significant association with dizziness. Each percentage point increase in weight loss was associated with a 6.3% increase in dizziness (OR = 1.063, 95% CI: 1.024–1.103, *p* = 0.001). No significant associations were observed for age (OR = 0.985, 95% CI: 0.946–1.027, *p* = 0.478), gender (OR = 1.414, 95% CI: 0.590–3.389, *p* = 0.437), supplement use (OR = 0.469, 95% CI: 0.101–2.191, *p* = 0.336), or time since surgery (OR = 1.220, 95% CI: 0.921–1.616, *p* = 0.166) (Table 3).

In the multivariate logistic regression model adjusting for age, gender, supplement use, and time since surgery, weight loss percentage showed significant association with dizziness (adjusted OR = 1.061, 95% CI: 1.017–1.107, *p* = 0.006). The multivariate model demonstrated acceptable fit (AIC = 143.546; pseudo R-squared = 0.083).

### 3.5. Associations Between Dizziness and Other Related Symptoms

Chi-square tests revealed significant associations between dizziness and other outcomes. Dizziness was strongly associated with imbalance (chi-square = 14.325, df = 1, *p* = 0.0002), with 46.6% of dizzy patients also reporting imbalance compared to only 8.8% of non-dizzy patients. Dizziness was also significantly associated with falls (chi-square = 7.085, df = 1, *p* = 0.008), with 25.9% of dizzy patients reporting falls compared to 2.9% of non-dizzy patients. The association between dizziness and hearing problems did not reach statistical significance (chi-square = 0.507, df = 1, *p* = 0.477) (Table 4).

**Figure 2:**
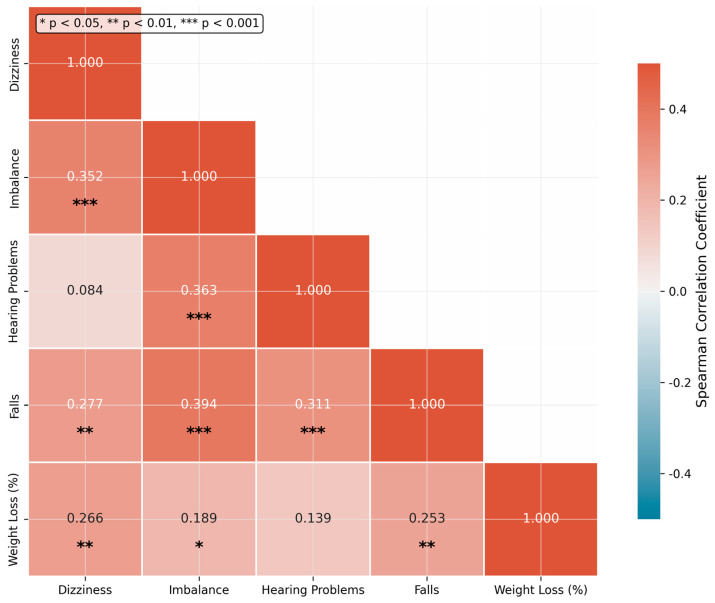
Spearman Correlations Between Vestibular Symptoms and Weight Loss (*N* = 136).

Spearman correlation analysis revealed moderate positive correlations between dizziness and imbalance (rho = 0.352, *p* = 0.0007), dizziness and weight loss percentage (rho = 0.266, *p* = 0.002), and imbalance and hearing problems (rho = 0.363, *p* = 0.0009). These correlations support the conceptualization of vestibular complications as an interconnected syndrome rather than isolated symptoms (Figure 2).

### 3.6. Treatment-Seeking Behavior

Despite the high prevalence of dizziness and its association with functional impairment, treatment-seeking behavior was notably limited. None of the 116 participants with dizziness reported taking specific medication for their symptoms, and no participants reported visiting a specialized balance clinic. This finding suggests a substantial gap in the recognition and management of vestibular complications following bariatric surgery.

## 4. Discussion

This cross-sectional study of 156 patients who underwent bariatric surgery reveals a strikingly high prevalence of post-operative dizziness and balance-related symptoms, with dizziness affecting over three-quarters of respondents, imbalance affecting nearly two in five, and hearing problems affecting approximately one in ten. The principal finding is the significant and independent association between the magnitude of weight loss and the occurrence of dizziness, with each percentage point of weight loss conferring a 6.1% increase was associated with experiencing symptoms. These findings have important implications for clinical practice and patient counseling in bariatric surgery programs.

The 77.3% prevalence of dizziness observed in our cohort substantially exceeds estimates from general population studies, which report point prevalence ranging from 15–42% [20]. This elevation warrants careful interpretation, reflecting both genuine post-surgical complications and methodological factors. Recruitment through clinics and support groups may have preferentially enrolled symptomatic individuals, while the broad definition of dizziness may have captured non-vestibular symptoms including orthostatic lightheadedness. The 77.3% figure should be considered an upper-bound estimate encompassing the full spectrum of post-operative dizziness-related symptoms rather than a precise measure of isolated vestibular dysfunction. This marked elevation suggests that bariatric surgery was associated with specific risk factors for vestibular dysfunction beyond those present in the general obese population. The temporal pattern of symptom onset, with the majority of first episodes occurring within the first 1–3 weeks post-surgery, implicates the immediate post-operative period as a critical window for vestibular symptom development. This timing coincides with the period of most rapid weight loss, hemodynamic adaptation, and nutritional transition, supporting the hypothesis that these physiological changes contribute to vestibular dysfunction.

The dose–response relationship between weight loss percentage and dizziness risk represents a novel finding with potential mechanistic implications. While previous studies have reported dizziness in individual patients following bariatric surgery, our data demonstrate a continuous gradient of risk across the spectrum of weight loss [21]. This pattern is consistent with several potential mechanisms. First, rapid weight loss may induce orthostatic hypotension through altered baroreceptor sensitivity and reduced cardiac preload, particularly during the early post-operative period when hemodynamic adaptation is most pronounced [22]. Second, hypoglycemic episodes related to altered glucose metabolism and early dumping syndrome may contribute to episodic lightheadedness and presyncope [23]. Third, nutritional deficiencies including vitamin B12, iron, and vitamin D may affect neural conduction and autonomic function [24]. Fourth, dehydration resulting from reduced oral intake and potential gastrointestinal losses may further exacerbate orthostatic symptoms [25]. Fifth, rapid changes in body mass may alter the mechanical properties of the vestibular system, including endolymphatic fluid dynamics [26].The strong association between dizziness and imbalance (OR = 14.2 in bivariate analysis) underscores the functional interconnectedness of these symptoms and their collective impact on post-operative recovery. The observation that 20.7% of participants experienced falls attributable to imbalance raises significant safety concerns, particularly given that falls represent a leading cause of injury-related morbidity and mortality in adults [27]. The combination of dizziness, imbalance, and falls creates a concerning triad that may limit the benefits of surgical weight loss by restricting mobility, reducing physical activity, and impairing quality of life.

The absence of significant associations between dizziness and demographic factors (age, gender), supplement use, or vitamin D deficiency was unexpected. Previous literature has suggested that female gender and older age may be risk factors for vestibular dysfunction, and nutritional deficiencies are frequently implicated in post-bariatric neurological complications [28]. The lack of association with supplement use may reflect the high prevalence of supplementation in our cohort (85.3%), potentially masking any protective effect through near-universal exposure. Alternatively, the specific formulations and compliance patterns of supplementation may be more important than binary use status.

The minimal treatment-seeking behavior observed among participants with dizziness is particularly concerning and suggests a systematic gap in the recognition and management of post-operative dizziness in bariatric surgery programs. Several factors may contribute to this observation. Patients may attribute their symptoms to expected post-operative adaptation and fail to report them to healthcare providers. Alternatively, healthcare providers may not systematically inquire about dizziness, orthostatic symptoms, or balance problems, or may lack awareness of their prevalence and significance. The absence of established screening protocols and referral pathways for evaluating dizziness (including orthostatic blood pressure assessment, glucose monitoring, and nutritional evaluation) in post-bariatric care may further contribute to underdiagnosis.

Our findings should be interpreted in light of several limitations. The cross-sectional design precludes definitive causal inference, and the observed prevalence of 77.3% should be interpreted cautiously. The recruitment strategy through clinics, support groups, and social media may have introduced selection bias, preferentially enrolling symptomatic individuals. Combined with response bias, this likely resulted in overrepresentation of affected individuals in our sample and we cannot exclude the possibility of recall bias in the self-reported assessment of symptoms and their timing. The absence of a control group of non-surgical obese individuals limits our ability to attribute symptoms specifically to surgical intervention versus underlying obesity-related factors. The absence of objective assessment for specific causes of dizziness (including orthostatic vital signs, glucose monitoring during symptomatic episodes, and comprehensive nutritional laboratory testing) represents a significant limitation. Without such measures, we cannot definitively distinguish between orthostatic hypotension, hypoglycemia, vestibular dysfunction, or other etiologies, nor can we quantify impairment severity. Self-reported symptoms may overestimate true pathology and the broad definition of dizziness used in this study likely captures heterogeneous symptoms with varying underlying mechanisms. Additionally, the reliance on self-reported symptoms without objective vestibular testing may result in misclassification, though the consistency of reported symptom patterns and their associations support the validity of our assessments.

From a clinical perspective, our findings support the implementation of systematic screening for dizziness and balance symptoms in post-bariatric care pathways. Given the high prevalence and early onset of symptoms, screening should commence in the immediate post-operative period and continue at regular intervals during the first year. Assessment should include orthostatic vital signs, evaluation for hypoglycemia, and review of nutritional supplementation. Patients reporting dizziness or imbalance should undergo structured assessment to characterize symptom patterns, identify associated features, and evaluate functional impact. Multidisciplinary management including internal medicine evaluation for orthostatic hypotension and metabolic causes, nutritional optimization, and referral to vestibular rehabilitation when indicated may be appropriate for patients with persistent or functionally limiting symptoms [29].

Future research should address the limitations of the current study through prospective cohort designs incorporating objective dizziness testing, detailed nutritional assessments, and longitudinal follow-up to characterize the natural history of dysfunction. Randomized trials evaluating preventive interventions, such as targeted supplementation protocols or rehabilitation programs, would provide valuable evidence for clinical practice. Additionally, mechanistic studies examining the physiological changes underlying vestibular symptoms following bariatric surgery would enhance our understanding of this underrecognized complication. Furthermore, the study primarily focused on patients who underwent sleeve gastrectomy and gastric bypass surgeries, which may limit the generalizability of the findings to other types of bariatric procedures. Additional research is necessary to determine whether different surgical methods are associated with varying levels of risk for dizziness and balance problems post-surgery. Participants’ self-reported experiences regarding dizziness and balance issues may be subject to memory bias, as individuals might not accurately recall or disclose their symptoms. Therefore, future research should consider incorporating objective measures for dizziness and balance, such as posturography or vestibular function assessments, to provide a more comprehensive evaluation of equilibrium issues within this population. The reliance on self-reported symptoms without clinical evaluation or objective vestibular testing limits diagnostic specificity. The questionnaire was designed specifically for this study as no validated instrument exists for post-bariatric vestibular symptoms. Future studies should consider incorporating standardized instruments such as the Dizziness Handicap Inventory alongside objective vestibular assessment.

## 5. Conclusions

In conclusion, post-surgery complications, particularly dizziness, are highly prevalent following bariatric surgery and demonstrate a significant dose–response relationship with the magnitude of weight loss. However, the 77.3% prevalence should be interpreted cautiously due to potential selection bias, response bias, and the broad definition of dizziness encompassing both vestibular and non-vestibular etiologies. The strong associations between dizziness and functional outcomes, including imbalance and falls, underscore the clinical importance of these symptoms. Enhanced awareness, systematic screening, and appropriate management integrated into comprehensive post-bariatric care to optimize patient outcomes and quality of life.

## Figures and Tables

**Table 1:** Demographic and Clinical Characteristics of Study Participants (*N* = 156).

Characteristic	*n* (%) or Mean ± SD
Age (years), mean ± SD	36.9 ± 9.9 (range: 18–65)
Female, *n* (%)	101 (64.7)
Male, *n* (%)	55 (35.3)
Gastric sleeve surgery, *n* (%)	144 (92.3)
Gastric bypass surgery, *n* (%)	7 (4.5)
Pre-operative weight (kg), mean ± SD	119.5 ± 24.6
Lowest post-operative weight (kg), mean ± SD	74.9 ± 17.4
Weight loss percentage (%), mean ± SD	36.2 ± 12.5
Time since surgery <1 year, *n* (%)	67 (42.9)
Time since surgery 1–3 years, *n* (%)	47 (30.1)
Time since surgery >3 years, *n* (%)	36 (23.1)
Supplement use, *n* (%)	133 (85.3)
Vitamin D deficiency, *n* (%)	68 (64.8)

Note: Percentage weight loss was calculated relative to pre-operative baseline weight using the formula: % weight loss = [(pre-operative weight − current weight)/pre-operative weight] × 100.

**Table 2:** Prevalence of Post-Operative Vestibular Symptoms (*N* = 150).

Symptom	*n*	*N*	Prevalence (%)	95% CI
Dizziness	116	150	77.3	70.0–83.3
Imbalance	57	150	38	30.6–46.0
Hearing Problems	16	150	10.7	6.7–16.6
Falls	31	150	20.7	15.0–27.8

Note: Six participants had missing data for all vestibular symptoms and were excluded from prevalence calculations.

**Table 3:** Logistic Regression Analysis: Predictors of Post-Operative Dizziness (*N* = 136).

Predictor	Bivariate OR (95% CI)	*p*-Value	Adjusted OR (95% CI) *	*p*-Value
Age (per year)	0.99 (0.95–1.03)	0.478	1.00 (0.95–1.05)	0.949
Gender (Male)	1.41 (0.59–3.39)	0.437	0.96 (0.37–2.50)	0.93
Weight loss (%)	1.06 (1.02–1.10)	0.001	1.06 (1.02–1.11)	0.006
Supplement use	0.47 (0.10–2.19)	0.336	0.75 (0.14–4.05)	0.738
Time since surgery	1.22 (0.92–1.62)	0.166	1.01 (0.74–1.39)	0.938

Note: *N* = 136 participants had complete data for all predictor variables. * Adjusted for age, gender, supplement use, and time since surgery.

**Table 4:** Chi-Square Tests for Associations Between Vestibular Symptoms (*N* = 150).

Association	Chi-Square	df	*p*-Value	Cramer’s V (95% CI)
Dizziness vs. Imbalance	14.325	1	0.0002	0.303 (0.150–0.443)
Dizziness vs. Falls	7.085	1	0.008	0.248 (0.090–0.393)
Dizziness vs. Hearing Problems	0.507	1	0.477	0.055 (0.000–0.213)
Imbalance vs. Falls	19.833	1	0.0004	0.380 (0.233–0.509)

## Data Availability

Data will be available upon request.

## Abbreviations

The following abbreviations are used in this manuscript:

BPPV	Benign paroxysmal positional vertigo
OR	Odds Ratio
CI	Confidence Interval
AIC	Akaike Information Criterion
BMI	Body Mass Index
SD	Standard Deviation
df	Degrees of Freedom
